# Peroneal artery pseudoaneurysm – a case report and literature review

**DOI:** 10.1186/1471-2482-7-4

**Published:** 2007-03-29

**Authors:** Umar Sadat, Teikchoon See, Claire Cousins, Paul Hayes, Michael Gaunt

**Affiliations:** 1 Cambridge Vascular Unit, Cambridge University Hospitals NHS Foundation Trust, Cambridge, UK; 2 University Department of Radiology, Cambridge University Hospitals NHS Foundation Trust, Cambridge, UK

## Abstract

**Background:**

Aneurysms of the peroneal artery are infrequent and consist mainly of pseudoaneurysms.

**Case presentation:**

This report describes an unusual case of peroneal pseudoaneurysm developing after thromoboembolectomy with a Fogarty catheter. It was managed successfully using an endovascular technique consisting of selective catheterization and coil embolization. The coils were placed in the peroneal artery, both proximal and distal to the pseudoaneurysm.

**Conclusion:**

Endovascular technique can be successfully used to treat pseudoaneurysms in difficult settings.

## Background

Pseudoaneurysms of the peroneal arteries being uncommon offer interesting management strategies [1] . An interesting case is presented and its management discussed below.

## Case presentation

A 68-year-old gentleman presented to the emergency department 2 weeks after thromboembolectomy for an acutely ischemic leg, with complaints of pain in the lower part of the right leg, both at rest and on walking. He was an ex-smoker with a history of multiple aneurysm repairs namely repair of a ruptured right common iliac aneurysm (2001), repair of a tender 4.5 cm left common femoral aneurysm (2005) and embolisation of a 3.9 cm left internal iliac aneurysm through a right common femoral access (2006). Just after the embolisation, he developed an acutely ischemic right leg, for which he underwent a successful thromoboembolectomy through a femoral arteriotomy. Embolectomy was performed using a size 2–3 Fogarty Embolectomy catheter. Fresh thrombus was retrieved and blood flow resumed with restoration of pre-event distal pulses.

The patient presented with right leg pain 2 weeks following embolectomy. On admission he had a pulse rate of 80 beats per minute and was normotensive. There was no apparent leg swelling, no visible sign of ischemia or injury to the leg. Dorsalis pedis pulse was not palpable though good posterior tibial and popliteal pulses were present. An expansile swelling was palpable on the anteromedial surface of the leg, 5 cm above the ankle.

**Figure 1:**
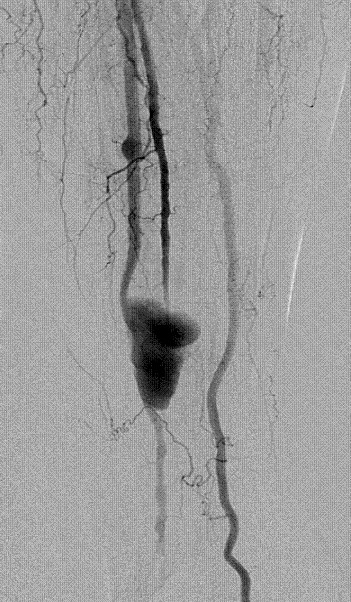
Distal subtraction angiogram shows a distal peroneal pseudoaneurysm and a mid peroneal aneurysmal dilatation.

**Figure 2:**
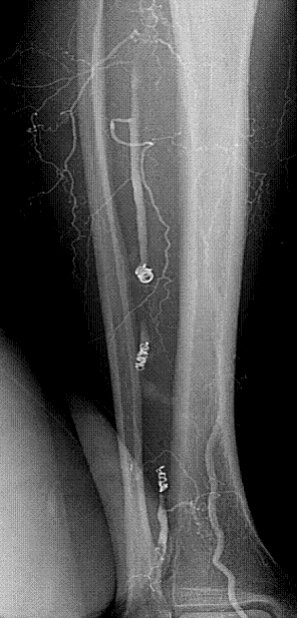
Post embolisation angiogram showing coils in-situ and no flow into the Pseudoaneurys.

A colour Doppler ultrasound of the leg suggested the presence of a peroneal pseudoaneurysm. Digital subtraction angiography confirmed a 5.1 × 2.4 cm pseudoaneurysm at the distal third of the mid peroneal artery. There was also a small aneurysmal dilatation of the mid peroneal artery (Figure 1). Embolization using five 4 mm × 2 mm coils was performed, proximal and distal to the pseudoaneurysm (Figure 2). The more proximal aneurismal dilatation was also embolised using two 5 mm × 5 mm coils (Figure 2). Post embolisation ultrasound 24 hrs later confirmed no flow into the pseudoaneurysm. The patient was symptom free and was discharged from hospital after 2 days.

## Discussion and conclusion

Aneurysms of the peroneal artery are infrequent and consist mainly of pseudoaneurysms. Thrombolembolectomy using a fogarty balloon catheter [1] and leg trauma (ankle sprain [2], penetrating or blunt injury [3,4], bimalleolar [5] fracture of the ankle) have been documented in literature as their main causes, besides mycotic [6] or connective tissue disorders such as Behcet's disease [7].

The vast majority of the reported cases of mycotic pseudoaneurysms involve major axial vessels proximal to popliteal artery but there has been rare citation of multiple tibioperoneal pseudoaneurysms [6]. Causes of mycotic aneurysms include infective endocarditis, most commonly caused by gram-positive pathogens in intravenous drug Abusers [8] however in a rare case brucella canis has also been documented as cause of multiple mycotic tibioperoneal aneurysms[6].

The pathogenesis of pseudoaneurysms is characterized by localized disruption of the arterial wall leading to the extravasation of the blood into surrounding tissue that becomes walled off by the surrounding layers of connective tissue. However they have a persistent channel communicating with the artery making them pulsatile. These can be asymptomatic or may present with leg swelling, bruising and pain or neurological signs due to nerve compression (more common in popliteal aneurysms), or rupture.

The management of pseudo aneurysms is varied. They can thrombose without any intervention as reported by Kocakoc and colleagues [9] however most are treated with radiological or surgical intervention. Endovascular options include coil embolization [1] as performed in our case, thrombin injection [10] or stent insertion [11]. Another option is proximal balloon occlusion to allow the pseudoaneurysm to thrombose.

Surgical management involves the evacuation of the haematoma after proximal and distal vascular control has been achieved. The defect in the arterial wall can either be repaired by primary closure or by insertion of a vein patch. Vein interposition graft or prosthetic graft can also be used if the segment of the disrupted artery cannot be primarily repaired. In our case endovascular coil embolization was appropriate because the posterior tibial artery was the dominant arterial supply to the foot and therefore the peroneal artery could be safely sacrificed. Surgical repair would have been preferred if a functioning peroneal artery was required to maintain a viable blood supply.

This patient presented two weeks after thromboembolectomy performed for an acutely ischemic right leg. The likely aetiology is a traumatic injury imposed by the Fogarty catheter. To prevent such complication, gentle manipulation with the Fogarty catheter is essential. Patients such as this with extensive aneurysmal disease clearly have arterial walls that are relatively easily traumatised, with defective collagen and elastin. A full review of the metabolic defects underlying aneurysm formation is beyond the scope of this case report. In addition, angiography or colour Doppler ultrasound is necessary to identify these complications, because the presence of distal pulses does not preclude complications such as perforation, longitudinal arterial tear or arteriovenous fistula formation [1].

The majority of femoral embolectomies are still performed "blind" as opposed to using a guidewire and radiological guidance. In the majority of cases the catheter will tend to pass down the peroneal artery as this has the straightest course down to the ankle from the popliteal artery. Care must be taken not to over inflate the balloon in the peroneal artery to avoid disrupting the integrity of the arterial wall and to prevent pseudoaneurysm formation, particularly in more vulnerable patients such as that described.

## Competing interests

The author(s) declare that they have no competing interests.

## Authors' contributions

All the authors have been involved in literature search, writing and final reviewing of this manuscript.

## Pre-publication history

The pre-publication history for this paper can be accessed here:


